# Distinct signal transductions in fast‐ and slow‐ twitch muscles upon denervation

**DOI:** 10.14814/phy2.13606

**Published:** 2018-02-21

**Authors:** Hongbo Gao, Yi‐Fan Li

**Affiliations:** ^1^ Division of Basic Biomedical Sciences Sanford School of Medicine University of South Dakota Vermillion South Dakota

**Keywords:** Denervation, EDL, signal transduction, soleus

## Abstract

Denervation induces skeletal muscle atrophy, which primarily impairs oxidative slow twitch fibers. The underlying mechanism of this phenomenon, however, remains to be addressed. We hypothesize that denervation‐induced fiber‐specific atrophy may result from the distinct activities of different signaling pathways that are involved in protein synthesis and degradation in fast‐ and slow‐twitch fibers. In this study, 1‐month‐old male mice were subjected to unilateral sciatic denervation for 4 days. Fast‐twitch muscle extensor digitorum longus (EDL) and slow‐twitch muscle soleus were collected from the denervated side and the control side of hind limbs. Total and phosphorylated protein levels of key factors of major signaling pathways in these tissues were determined using western blot assay. Our data showed that total AKT and FoxO3 protein levels were upregulated in denervated muscles as compared with control sides. Phosphorylation of AKT and FoxO3 were proportionally enhanced in denervated EDL but not soleus, indicating AKT activation drives phosphorylation of FoxO3 in EDL but not in soleus upon denervation. As a result, FoxO3‐targeted atrogenes MurF1 and Atrogin1 protein abundances were reduced in denervated EDL but not altered in soleus. In consistent with this change, polyubiquitination were significantly increased in denervated soleus, but only a slight increase in ubiquitination was found in denervated EDL. Autophagy marker LC3 protein level was significantly increased in both muscle types, but in greater extent in EDL after denervation. IRS1 protein level and active ERK were reduced in both muscles upon denervation, which might contribute to the upregulation of total AKT protein level and FoxO3 abundance in EDL and soleus. Total and phosphorylated AMPK protein levels were increased in denervated soleus but not in EDL. Overall, these data reveal that the key signaling pathways that regulate protein synthesis and degradation are more sensitive in soleus than EDL in response to denervation.

## Introduction

Skeletal muscle composes distinct muscle fibers, which are defined by myosin heavy chain (MyHC) isoforms. There are four types of MyHC specified by their distinct metabolic activities. Type I is the slow‐twitch oxidative fiber, type IIa is the fast‐twitch oxidative fiber, type IIx/d and type IIb are fast‐twitch glycolytic fibers. Soleus is the typical muscle with slow‐twitch fibers, and extensor digitorum longus (EDL) is predominantly composed of fast‐twitch glycolytic fibers (Schiaffino and Reggiani 2011). Skeletal muscle denervation, such as in trauma and aging, causes muscle atrophy, denervation‐induced muscle atrophy features greater extent of muscle loss in type I fiber than type II type (Macpherson et al. 2011; Wang and Pessin 2013). The underlying mechanism by which type I fiber is more prone to atrophy remains to be fully understood. A thorough understanding of this mechanism is critical for development of a strategy to prevent the detrimental consequences of denervation‐induced muscle atrophy.

Muscle atrophy, characterized as loss of contractile proteins, especially decrease in MyHC, is the consequence of the decline of protein synthesis and enhanced protein degradation. Insulin‐like growth factor (IGF)‐AKT pathway is crucial in regulation of protein synthesis and degradation in skeletal muscles (Singleton et al. 2000). The activation of AKT will upregulate protein synthesis, and meanwhile, inhibit protein degradation through phosphorylation of Forkhead‐box type O 3 (FoxO3). As a transcriptional factor (Sandri et al. 2004). FoxO3 regulates protein degradation in skeletal muscle via upregulation of ubiquitin E3 ligases, like muscle RING‐finger protein‐1 (MurF1) and Atrogin1 (Bodine and Baehr 2014). The phosphorylation of FoxO3 at Ser253 by AKT blocks it to enter nucleus and execute transcriptional activity (Sandri et al. 2004). FoxO3 also promotes macroautophagy‐related genes expression in skeletal muscles, including microtubule‐associated protein 1A/1B‐light chain 3 (LC3) (Zhao et al. 2007). Additionally, AMP‐activated protein kinase (AMPK) enhances FoxO3 transcriptional activity when FoxO3 is translocated into nucleus (Greer et al. 2007), whereas peroxisome‐proliferator‐activated receptor‐*γ* coactivator‐1 alpha (PGC1*α*) inhibits FoxO3 function (Sandri et al. 2006). Interestingly, activated AMPK stimulates PGC1*α* gene expression in response to endurance exercise (Misu et al. 2017). It is still unclear whether activation and interaction of these signal transductions are distinct between slow‐ and fast‐ twitch fibers upon denervation. In this study, we compared the activation of these signal factors from two kinds of fibers to understand if the specific activation of a signaling pathway occurs in a fiber‐dependent manner.

## Methods

### Sciatic denervation

C57/black6 male mice at age of 1 month old were used. The animal use and the experimental protocol were reviewed and approved by the University of South Dakota Institutional Animal Care and Use Committee (IACUC), and were in compliance with the NIH guideline. The unilateral sciatic denervation was performed by following the published method (MacDonald et al. 2014). Briefly, animals were anesthetized using 2% isoflurane, the dorsal hind limb of the denervation side was shaved and disinfected using betadine and 75% ethanol scrub. A small incision was made through the skin, the sciatic nerve was isolated from adjacent tissues, sectioned, and 2 mm of sciatic nerve was removed. The incision was closed using a surgical adhesive. After 4 days of denervation, under isoflurane anesthesia, EDL and soleus were isolated from denervation side and control side, snap frozen, and stored at −75°C for western blots. Tibialis anterior (TA) were isolated from both sides for muscle weight.

### Western blot

Muscle tissues were homogenized in RIPA buffer containing a protease inhibitor cocktail (Santa Cruz, Dallas, TX) and phosphatase inhibitor (Research Product International, Mount Prospect, IL). Protein concentration of samples were determined by a standard BCA assay and the samples were subjected to standard western blot protocol, as described previously (Hartnett et al. 2015). Proteins that were transferred onto the membranes were immunoblotted using the following primary antibodies: AKT, phosphorylated AKT (Ser473), FoxO3, phosphorylated FoxO3 (Ser253), LC3, IRS1, AMPK*α* and phosphorylated AMPK*α* (Thr172) (Cell Signaling Technologies, Danvers, MA); GAPDH, Ubiquitin, pERK and ERK (Santa Cruz Inc., Santa Cruz, CA); MurF1 and Atrogin1 (GeneTex, Irvine, CA); and PGC1*α* (Millipore‐Sigma, St. Louis, MO). The secondary antibodies conjugated with Alex‐700 or ‐800 fluorescence were purchased from Invitrogen (Thermo Fisher Scientific Inc., Carlsbad, CA). The fluorescent signals of the blots were detected by LI‐COR scanner (LI‐COR Biosciences, Lincoln, NE) and quantified using LI‐COR Image Studio.

### Data analysis

Raw data calculation, graphing, and descriptive statistics were performed using Microsoft Excel Data Analysis package. Data were presented as Mean ± Standard Deviation (SD). Statistical significance between denervated muscle and control sides, and EDL versus soleus were compared by two‐factor analysis of variance (ANOVA) followed by unpaired two‐tailed Student's *t*‐test. Statistical significances were determined by *P* value less than 0.05.

## Results

### AKT was activated in EDL but not in soleus upon denervation

Denervation‐induced muscle atrophy was detectable at 4th day of postsurgery, evidenced by 15% of muscle weight loss in tibialis anterior (TA) as compared with contralateral TA (*P *=* *0.03).

**Figure 1:**
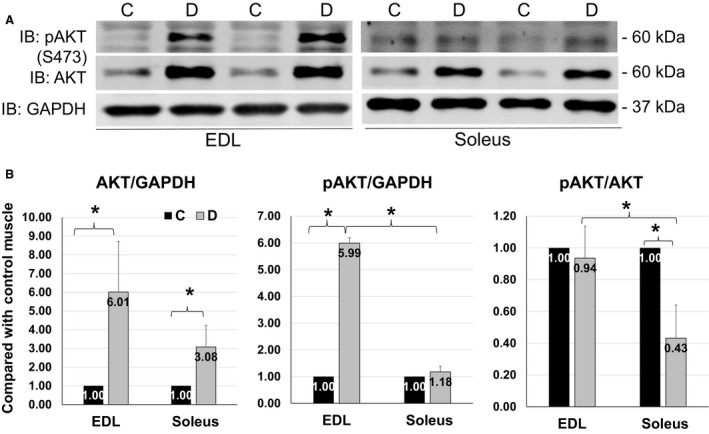
Distinct AKT activation in EDL and soleus upon denervation. (A) Representative images of western blots for pAKT (S473), AKT and GAPDH from EDL (left) and soleus (right) on day 4 of sciatic denervation. (B) Quantifications of western blots for total AKT and phosphorylation of AKT (S473) from EDL and soleus, expressed as fold increase in denervated muscles versus control ones. Data are presented as mean ± SD. “*” indicates significant difference between two groups (*P *<* *0.05), *n* = 4 (C, control; D, denervation).

Total AKT protein abundance was enhanced in denervated muscles, with a sixfold increase in EDL but only a threefold increase in soleus in response to denervation (Fig. 1B left panel). More interestingly, phosphorylation of AKT was significantly enhanced in EDL but was negligible in soleus upon denervation, as compared with control (Fig. 1B middle panel). As a result, the ratio of phosphorylated AKT versus total AKT was significantly reduced in denervated soleus (Fig. 1B right panel). Activation of AKT pathway during denervation is likely a compensatory response that can alleviate atrophy. Therefore, lack of AKT activation in soleus may partially explain the vulnerability of slow‐twitch fibers in denervation condition.

### Changes of total and phosphorylated FoxO3 were different in EDL and soleus upon denervation

**Figure 2:**
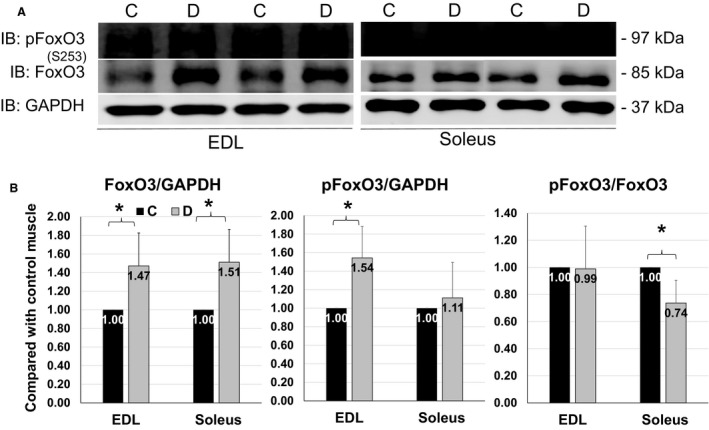
FoxO3 phosphorylation in EDL and soleus. (A) Representative images of western blots for pFoxO3(S253), FoxO3 and GAPDH from EDL (left) and soleus (right) after 4 days of denervation. (B) Quantifications of western blots for total FoxO3 and pFoxO3 from EDL and soleus. Data are in mean ± SD. “*” indicates significant difference between two groups (*P *<* *0.05), *n* = 4 (C, control, D, denervation).

FoxO3 is a negative regulator for the maintenance of muscle mass, which is mediated by its transcriptional activity. FoxO3 combines with the promoters of target genes to upregulate atrogenes expression. The phosphorylation of FoxO3, especially when phosphorylated by AKT at Ser253 residue, blocks its translocation from cytosol to nucleus (Santo et al. 2013) (summarized in Fig. 7A). We found that total FoxO3 was upregulated in EDL and soleus by 1.5‐fold in response to denervation (Fig. 2B left panel). However, phosphorylation of FoxO3 at Ser253 was only significantly increased in denervated EDL but not in soleus (Fig. 2B middle panel). As a result, the ratio of pFoxO3 to total FoxO3 was significantly reduced in soleus (Fig. 2B right panel). This is likely the result of the weak AKT activation in denervated soleus as shown in Figure 1, because phosphorylation at Ser253 in FoxO3 is mediated by AKT. Given the fact that Ser253 phosphorylation inhibits FoxO3 to enter nucleus, this result suggests a potential greater FoxO3 nuclear translocation and upregulation of atrogenes in denervated soleus than in EDL, which contributes to the vulnerability of soleus to denervation.

### Protein levels of MurF1 and Atrogin1 were reduced in EDL but unchanged in soleus

**Figure 3:**
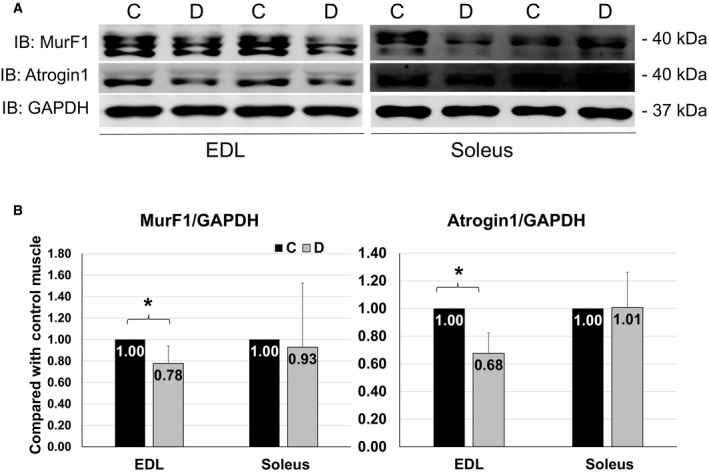
MurF1 and Atrogin1 protein levels in muscles. (A) Representative images of western blots for MurF1, Atrogin1 and GAPDH from EDL (left) and soleus (right) in 4 days of denervation. (B) Quantifications of western blots for MurF1 and Atrogin1 from EDL and Soleus. Data are in mean ± SD. “*” indicates significant difference between two groups (*P *<* *0.05), *n* = 4 (C, control, D, denervation).

MurF1 and Atrogin1 are the atrogenes that are regulated by FoxO3 in skeletal muscle (Fig. 7A). MurF1 and Atrogin1 function as ubiquitin E3 ligases that cause polyubiquitination and proteasome‐mediated degradation of muscular contractile proteins, like MyHC and *α*‐Actinin (Bilodeau et al. 2016). Most of studies use RT‐PCR showing that FoxO3 upregulates MurF1 and Atrogin1 mRNA levels during muscle atrophy but few is done in assessing MurF1 and Atrogin1 protein levels in denervation. Our data showed that MurF1 and Atrogin1 protein levels were reduced in EDL (Fig. 3B left panel) but unchanged in soleus at day 4 of denervation (Fig. 3B right panel). At this time, it is not known why the protein levels of these two E3 ligases were downregulated in denervated EDL. It is possible that the protein turnovers (degradation) of these E3 ligases are also increased as a compensatory mechanism trying to mitigate atrophy during denervation. This response is likely greater in EDL but weaker in soleus. This is consistent with the greater activation of AKT and phosphorylation of FoxO3 in EDL as shown in Figures 1 and 2. This suggests that at the early stage of denervation, the compensatory protective effect against atrophy mediated by AKT pathway is greater in EDL and weaker in soleus, resulting in a greater susceptibility of soleus to muscle wasting.

### Polyubiquitination was increased in soleus, and enhancement of LC3 was more remarkable in EDL

**Figure 4:**
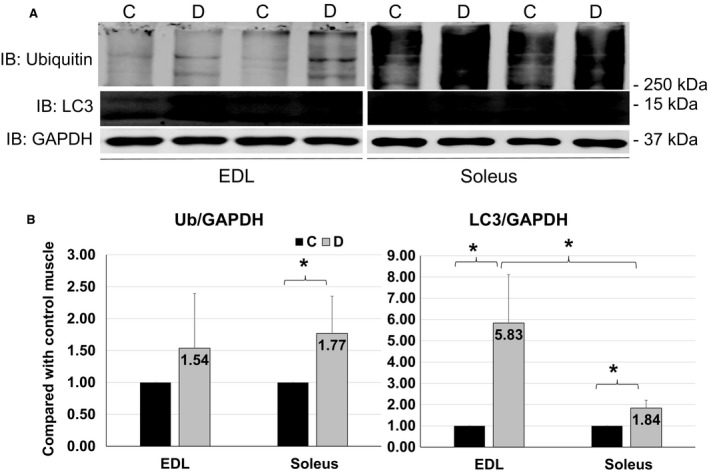
Polyubiquitination and LC3 levels. (A) Representative images of western blots for ubiquitin, LC3 and GAPDH from EDL (left) and soleus (right) in 4 days of denervation. (B) Quantifications of Western blots for polyubiquitin chain and LC3 from EDL and Soleus. Data are in mean ± SD. “*” indicates significant difference between two groups (*P *<* *0.05), *n* = 4 (C, control, D, denervation).

Consistent with the greater E3 ligases levels in denervated soleus than EDL as shown in Figure 3, the level of polyubiquitination was significantly increased in soleus but not in EDL upon denervation (Fig. 4B left panel). On the other hand, a greater increase in LC3 protein (sixfold increment) was found in EDL than soleus (only 1.8‐fold enhancement) from denervation sides as compared with control muscles (Fig. 4B right panel). These data confirm that lack of AKT activation leads to greater polyubiquitination‐mediated protein degradation in denervated soleus. Whether the activation of autophagy is protective or detrimental and what is the functional role of greater autophagy activation in fast‐twitch muscles during denervation need to be further tested.

### IRS1 abundance and ERK activity were reduced in denervated EDL and soleus as compared with control muscles

**Figure 5:**
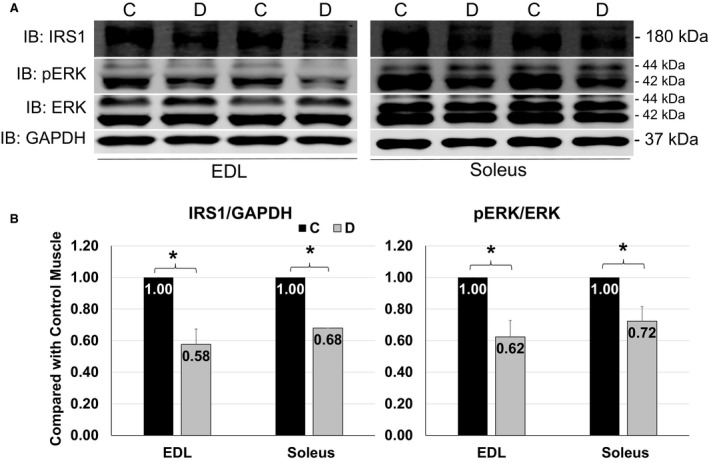
IRS1 abundance and ERK activation in muscles. (A) Representative images of western blots for IRS1, pERK and ERK and GAPDH from EDL (left) and soleus (right) at 4 days of denervation. (B) Quantifications of western blots for IRS1 and ERK activity from EDL and Soleus. Data are in mean ± SD. “*” indicates significant difference between two groups (*P *<* *0.05), *n* = 4 (C, control, D, denervation).

To seek the potential explanation for the elevation of AKT and FoxO3 protein levels in denervated EDL and soleus, we measured the level of insulin receptor substrate 1 (IRS1), an upstream protein of AKT (Wu et al. 2011), and tested the activation of extracellular signal‐regulated kinases (ERK) that regulates FoxO3 degradation in skeletal muscles (Yang et al. 2008). IRS1 protein levels were significantly reduced in denervated EDL and soleus (Fig. 5B left panel). The decline of IRS1 in denervated muscles might contribute to upregulation of AKT total protein levels, because IGF‐IRS1 signaling pathway mediates phosphorylation of AKT at Ser473, which marks AKT to be degraded by ubiquitin proteasome system (UPS) (Wu et al. 2011). Downregulation of IRS1 reduced the potency of AKT phosphorylation (Machado‐Neto et al. 2011). Meanwhile, IRS1 also transduces signal into ERK, and IRS1 deficiency reduces ERK phosphorylation (Machado‐Neto et al. 2011) (Fig. 7A). Consistently, our data show that ERK phosphorylation was significantly declined in denervated EDL and soleus (Fig. 5B right panel). This reduction in ERK activity may contribute to the upregulation of FoxO3 because activated ERK phosphorylates FoxO3 at Ser294, Ser344, and Ser425, which promotes FoxO3 degradation via UPS (Yang et al. 2008). Therefore, the upregulation of AKT and FoxO3 protein levels might be resulted from lower level of IRS1 and ERK activities, and the inhibition of ERK activities might also be induced by IRS1 deficiency in denervated muscles.

### AMPK total protein level and activity were upregulated in soleus after denervation, and PGC1*α* abundance was enhanced in both EDL and soleus

**Figure 6:**
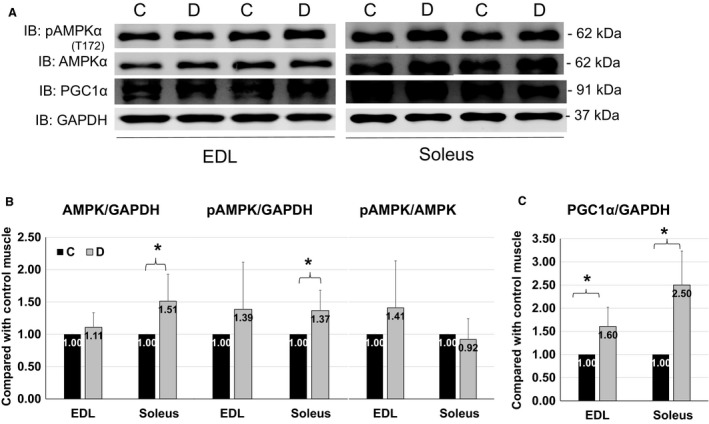
Total and phosphorylated AMPK protein levels and PGC1*α* abundance in muscles. (A) Representative images of western blots for pAMPK, AMPK, PGC1*α* and GAPDH from EDL (left) and soleus (right) in 4 days of denervation. (B) Quantifications of western blots for total and phosphorylated AMPK protein levels from EDL and Soleus. (C) Quantification of Western blots for PGC1*α* abundance from two types of muscles. Data are in mean ± SD. “*” indicates significant difference between two groups (*P *<* *0.05), *n* = 4 (C, control, D, denervation).

**Figure 7:**
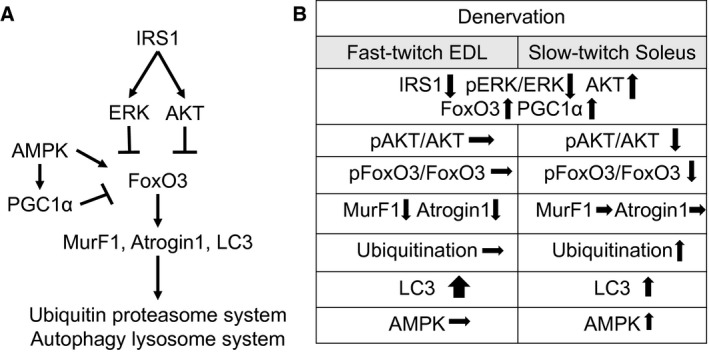
Summary of responses of signaling pathways in two types of muscles upon denervation. (A) Schematic illustration of major pathways that are involved in regulation of protein degradation in muscle. “**↓**” indicates stimulatory effect; “┴” indicates inhibitory effect. (B) Summary of the measurements of major signaling molecules in EDL and soleus with denervation. “↑” “↓” and “→”, respectively, indicate increased, decreased, and unchanged.

AMPK (Greer et al. 2007) and PGC1*α* (Sandri et al. 2006) are involved in the regulation of FoxO3 activities in muscle atrophy. Meanwhile, AMPK‐PGC1*α* axis improves muscle endurance via enhancing mitochondrial content (Pilegaard et al. 2003; Russell et al. 2003), and activated AMPK increases PGC1*α* protein level and activities (Iwabu et al. 2010). PGC1*α* is pivotal for type I myofiber formation and protects slow oxidative fibers from atrophy (Lin et al. 2002). The total protein and phosphorylation levels of AMPK were not altered in denervated EDL, but enhanced in soleus after denervation (Fig. 6B). PGC1*α* was upregulated in both muscle fibers upon denervation, but with a trend of greater increment in denervated soleus (2.5‐fold) than EDL (1.6‐fold) (Fig. 6C). Since activated AMPK enhances FoxO3 activity (Greer et al. 2007) and PGC1*α* inhibits FoxO3 function (Sandri et al. 2006) (Fig. 7A), upregulation of AMPK in soleus potentiates slow‐twitch fiber loss in denervation, and the specific greater increment in PGC1*α* in soleus might be a compensatory response to ameliorate denervation‐induced slow‐twitch fiber loss.

### Summary of different signaling activation in fast EDL and slow soleus upon 4 days of denervation

During denervation, the consistent molecular events occurring in both EDL and soleus muscles were the upregulation of total protein levels of AKT, FoxO3, and PGC1*α* as well as the downregulation of IRS1 protein level and ERK phosphorylation (Fig. 7B). The distinct changes of signal transduction in two types of muscles upon denervation include: AKT and FoxO3 phosphorylation were reduced in soleus but unaltered in EDL; protein levels of MurF1 and Atrogin1 were reduced in EDL but not in soleus; polyubiquitination was upregulated in soleus but not altered in EDL, while the increment of LC3 was more remarkable in EDL than in soleus; AMPK total protein and phosphorylation levels were upregulated in soleus but not changed in EDL; and PGC1*α* was upregulated in fast‐ and slow‐ twitch muscles (Fig. 7B). Overall, the distinct responses of those signaling transduction imply greater sensitivity and vulnerability of slow‐twitch fibers to denervation.

## Discussion

Denervation enhances muscle protein degradation, leading to the loss of muscular contractile proteins, characterizing as an initial rapid (first 2 weeks) and then slow (following 2 weeks) phase (Sacheck et al. 2007). Responses to sciatic nerve section are different in two types of muscles, with type I slow‐twitch fibers being more sensitive to denervation as compared with type II fast fibers (Wang and Pessin 2013). The underlying mechanisms of this difference, however, remain to be fully understood. IGF‐AKT pathway is crucial to regulate muscle growth (Pallafacchina et al. 2002) as well as protein degradation via phosphorylation of FoxO3 (Sandri et al. 2004). Hence, we compared AKT and FoxO3 activation in two types of muscles, fast twitch muscle EDL and slow twitch muscle soleus, after 4 days of denervation. Our study identifies the distinct responses of these signaling pathways in these two different muscle types to denervation. AKT is significantly activated in EDL at 4‐day denervation. We interpret this as a compensatory response in attempt to mitigate muscle injury and loss. This AKT activation is noticeably absent in slow‐twitch muscle, which was followed by a lower level of FoxO3 phosphorylation and a greater polyubiquitination in soleus as compared with EDL. These data provide novel information that can at least partially explain at signaling transduction level as why slow‐twitch muscles are more susceptible than fast‐twitch muscles in denervation‐induced atrophy. AKT signaling pathway enhances glucose uptake in skeletal muscles by promoting glucose transporter plasma membrane translocation of type 4 glucose transporter (Glut 4). The different levels of AKT phosphorylation in two types of muscles can also explain the previous observation that the basal level of 2‐deoxyglucose uptake was remarkably elevated in fast‐twitch fibers, but reduced in soleus as compared with control muscles after 3 days of denervation (Turinsky and Damrau‐Abney 1998).

FoxO3 is the transcriptional factor that regulates Atrogin1 and MurF1 gene expression in skeletal muscles. Atrogin1 and MurF1 are major E3 ligases that are responsible for the increased ubiquitination and degradation of contractile proteins in denervation‐induced muscle atrophy (Sandri et al. 2004). According to literature, increased mRNA levels of Atrogin1 and MurF1 reached the peak at 3 days of denervation, and returned to the base line at 14 days (Sacheck et al. 2007). On the other hand, little is known about protein changes of these two E3 ligases during denervation. Our data showed that the protein levels of these two E3 ligases were reduced in denervated EDL but unchanged in soleus upon 4 days of denervation. The different changes in mRNA levels and protein levels of these E3 ligases in denervated muscles need further investigation. Nonetheless, the different changes of MurF1 and Atrogen1 protein levels in soleus and EDL were explainable by the changes of AKT activation in our study: in denervated EDL, increased AKT activation causes increased FoxO3 phosphorylation, resulting in a reduced expression of these two E3 enzymes. In contrast, the absence of AKT activation and unchanged FoxO3 phosphorylation can explain the stable level of MurF1 and Atrogen1 in denervated soleus. Overall, these data suggest that due to the lack of AKT activation, ubiquitination‐mediated protein degradation will be more active in soleus in denervation.

In addition to UPS, protein degradation in skeletal muscles is also dependent on autophagy‐lysosome system. It is evident that these two degradation mechanisms may respond differently in fast‐ and slow‐twitch muscles. For instance, EDL is more sensitive to starvation‐induced atrophy with marked upregulation of macroautophagy (Yamada et al. 2012). We found that in denervated EDL, polyubiquitination level was unchanged but LC3 level was significantly increased, which contrasted with the significantly increased polyubiquitination and only moderately increased LC3 in denervated soleus. These data suggest the different preferences of protein degradation mechanisms in these two types of muscle. Autophagy may be more sensitive in fast‐twitch muscles, while UPS may play a greater role in slow‐twitch muscle in denervation‐induced atrophy. Our study is consistent with previous reports that LC3 positive autophagosome was more robust in EDL than in soleus under nutrient deprivation (Mizushima et al. 2004; Tanida et al. 2008). By comparing the responses of UPS and autophagy in fast‐ and slow‐twitch muscle to denervation, our data provide novel evidence indicating that the selection of protein degradation by UPS or autophagy mechanisms depends on muscle types upon denervation.

The upregulation of total protein levels of AKT and FoxO3 were noticed in both EDL and soleus muscles. The underlying mechanisms of these changes remain to be further addressed. In this study, reductions in IRS1 protein level and ERK phosphorylation were observed in both EDL and soleus after denervation. IRS1 mediates phosphorylation‐dependent ATK degradation. Depletion of IRS1 inhibits AKT and ERK activities (Machado‐Neto et al. 2011), and lack of phosphorylation at Ser473 residue protects AKT from UPS‐mediated degradation (Wu et al. 2011). Therefore, enhancement of AKT total protein level in denervation could be related to the reduction in IRS1‐facilitated AKT degradation. Moreover, activated ERK phosphorylates FoxO3 to promote UPS‐mediated FoxO3 protein degradation (Yang et al. 2008). The reduction in ERK activity observed in both denervated EDL and soleus might result in the less degradation of FoxO3 in denervated muscles.

Besides AKT, AMPK is another crucial factor in regulation of FoxO3. AMPK is the energy sensor in response to shortage of energy that promotes AMPK activation via phosphorylation at Thr172 residues. The active AMPK will stimulate catabolic process and inhibit anabolic process, including enhancement of protein degradation and inhibition of protein synthesis (Greer et al. 2007; Goodman et al. 2011). AMPK promotes muscle atrophy through activation of FoxO3 and elevation of Atrogin1 expression (Greer et al. 2007). Our data show that denervation did not alter AMPK abundance or activity in EDL, but total and phosphorylated AMPK protein levels were upregulated in denervated soleus, suggesting the sensitivity of slow‐twitch fibers to denervation might be partially caused by upregulation of AMPK function.

Active AMPK in skeletal muscles can upregulate PGC1*α* expression and enhance PGC1*α* transcriptional function through phosphorylation and deacetylation (Iwabu et al. 2010). PGC1*α* stimulates mitobiogenesis and improves mitochondrial function in response to endurance exercise (Pilegaard et al. 2003). Additionally, PGC1*α* inhibits FoxO3‐mediated atrogenes expression (Sandri et al. 2006). Our data show that the protein levels of PGC1*α* were upregulated in both types of muscles after denervation but the greater increase in PGC1*α* protein level was present in soleus, which was consistent with the upregulation of AMPK activities in soleus. AMPK‐PGC1*α* pathway may selectively serve as a compensatory mechanism in soleus, in contrast to EDL that primarily uses AKT as compensatory mechanism during atrophy. Denervation is reported to cause muscle oxidative stress, evidenced by high level of reactive oxygen species (ROS) (Karam et al. 2017). ROS in turns upregulates PGC1*α* expression via AMPK (Irrcher et al. 2009). As oxidative muscles, slow twitch muscles may have greater oxidative stress and exhibit greater upregulation of PGC1*α* as a compensatory response to denervation.

Conclusively, this study provides novel data that suggest the distinct activation of signal pathways in fast‐ and slow‐twitch muscles during denervation, which determines the different protein degradation mechanisms and discrete compensatory responses in specific muscle fiber types. Specifically, fast‐twitch muscles have greater AKT medicated compensatory response that may mitigate UPS‐mediated protein degradation. Additionally, autophagy is more sensitive in EDL. Slow‐twitch muscles lack AKT activation upon denervation, which lead to greater UPS mediated protein degradation, whereas greater PGC1*α* activation may serve as an alternative compensatory mechanism in slow muscles during denervation. Overall this novel information expands our understanding of the underlying mechanism of denervation‐induced muscle atrophy to explore new strategies to prevent and treat denervation and disuse‐related muscle atrophy.

## Conflict of Interest

None of the authors has any conflict of interest to disclose.
